# Outcomes of Dose Escalation of Imatinib in Chronic Myeloid Leukemia Patients: A Retrospective Analysis From an Indian University Teaching Hospital

**DOI:** 10.7759/cureus.70622

**Published:** 2024-10-01

**Authors:** Rajan Yadav, Harsha Panchal, Apurva Patel, Sonia Parikh, Kajal Shah

**Affiliations:** 1 Medical Oncology, Gujarat Cancer &amp; Research Institute (GCRI) and B J Medical College (BJMC), Ahmedabad, IND

**Keywords:** chronic myeloid leukemia (cml), dose escalation, imatinib mesylate, low and middle country (lmic), progression-free survival (pfs)

## Abstract

Background: Chronic myeloid leukemia (CML) treatment in low- and middle-income countries faces significant financial and logistical constraints. In scenarios where second-line tyrosine kinase inhibitors (TKIs) are unavailable or unaffordable, dose escalation of imatinib provides an alternative. This study evaluates the efficacy, safety, and progression-free survival (PFS) outcomes of dose escalation of imatinib in CML patients who experienced suboptimal response or progression on standard doses.

Methods: A retrospective analysis of 123 CML patients treated at an Indian university teaching hospital from 2013 to 2016 was conducted. Patients who showed progression on a 400 mg dose of imatinib were escalated to 600 mg, and further to 800 mg if required. Demographic data, progression, and toxicity were analyzed.

Results: Out of 123 patients, 78 (63.4%) showed a complete hematologic response after dose escalation. The median PFS was 48 months, with a three-year PFS rate of 67%. Notable toxicities included Grade 3/4 neutropenia in 15% and gastrointestinal disturbances in 12%. Comparatively, studies suggest that switching to a second-line TKI in similar settings results in a higher PFS; however, our findings underscore that dose escalation of imatinib remains a viable alternative when financial constraints limit access to second-line therapies.

Conclusion: In resource-constrained settings, dose escalation of imatinib can be an effective strategy for managing CML patients who progress on standard doses.

## Introduction

Chronic myeloid leukemia (CML) is a myeloproliferative disorder characterized by the presence of the BCR-ABL fusion gene, resulting from a translocation between chromosomes 9 and 22 (Philadelphia chromosome) [1]. Imatinib, a first-generation tyrosine kinase inhibitor (TKI), revolutionized the treatment of CML by targeting the BCR-ABL protein, leading to significant improvements in survival rates [2]. However, treatment options for patients who exhibit suboptimal response or resistance to standard-dose imatinib (400 mg) are limited, particularly in low- and middle-income countries (LMICs) where financial and logistical constraints often prevent access to second-line TKIs like dasatinib or nilotinib [3].

In this context, dose escalation of imatinib to 600 mg or 800 mg presents a viable alternative. Previous studies have demonstrated that higher doses of imatinib can overcome resistance in some patients, leading to improved clinical outcomes [4]. Nevertheless, the efficacy and safety of this approach remain underreported, especially in resource-constrained settings [5].

This study aims to evaluate the outcomes of imatinib dose escalation in CML patients treated at an Indian university teaching hospital. We hypothesized that dose escalation could provide a feasible and effective strategy for managing CML in patients who are unable to access second-line therapies due to financial or logistical constraints.

## Materials and methods

Study design and population

This retrospective study was conducted at an Indian university teaching hospital, analyzing data from 123 patients diagnosed with CML between 2013 and 2016. All patients included in the study were initially treated with imatinib 400 mg daily. Patients who exhibited suboptimal response or disease progression, defined according to European LeukemiaNet (ELN) criteria [6], were escalated to 600 mg daily, with further escalation to 800 mg daily if needed.

Data collection

Demographic information, including age, gender, and disease phase at diagnosis (chronic, accelerated, or blast phase), was collected from medical records. Progression data, including time to progression and response rates, were also recorded. Toxicity data were collected and graded according to the Common Terminology Criteria for Adverse Events (CTCAE) version 4.0 [7].

Statistical analysis

Progression-free survival (PFS) was defined as the time from the start of imatinib treatment to disease progression or death from any cause. PFS was estimated using the Kaplan-Meier method, and comparisons between groups were made using the log-rank test [8]. A p-value of less than 0.05 was considered statistically significant.

## Results

Patient demographics and baseline characteristics

The study included 123 patients with a confirmed diagnosis of CML. The median age at diagnosis was 45 years (range: 18-70 years). The majority of patients were male (63%). At diagnosis, 85% of the patients were in the chronic phase, 10% in the accelerated phase, and 5% in the blast phase [9] (Table 1).

**Table 1:** Baseline Demographic Characteristics

Characteristic	Value
Median Age (years)	45
Age Range (years)	18-70
Male (%)	63
Chronic Phase (%)	85
Accelerated Phase (%)	10
Blast Phase (%)	5
Median Hemoglobin (g/dL)	11.5
Median Platelet Count (x10^9^/L)	280

Response to dose escalation

Following dose escalation to 600 mg of imatinib, 63 patients (51%) achieved a complete hematologic response (CHR). An additional 15 patients (12%) required further escalation to 800 mg to achieve CHR. The overall CHR rate after dose escalation was 63.4% (78 out of 123 patients) [10]. Table 2 details the response rates at each dose level.

**Table 2:** Dose Escalation CHR, complete hematologic response.

Imatinib Dose (mg)	Patients Achieving CHR	Percentage	Patients Not Achieving CHR
400	40	67.5	83
600	23	48.8	60
800	15	36.6	45

The doses of imatinib ranged from 400 mg to 800 mg. Forty patients (32.5%) achieved a CHR on 400 mg of imatinib. After escalation to 600 mg, an additional 23 patients (18.7%) achieved CHR, and further escalation to 800 mg brought 15 more patients (12.2%) to CHR.

Progression-free survival

**Figure 1:**
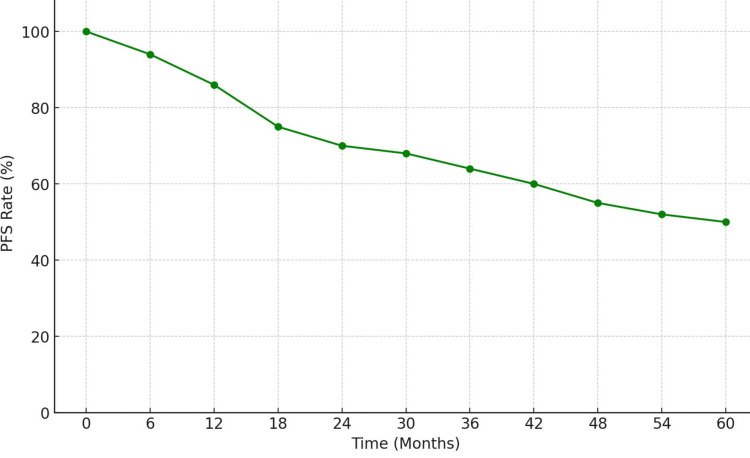
Progression-Free Survival PFS, progression-free survival.

The median PFS for the entire cohort was 48 months (95% CI: 42-54 months). The three-year PFS rate was 67%. Patients who responded to dose escalation had a significantly longer PFS compared to those who did not respond (median PFS: 56 months vs. 28 months, p < 0.01) [11]. Figure 1 shows the Kaplan-Meier curve for PFS stratified by response to dose escalation.

Toxicity

Toxicity profiles were consistent with previous reports of imatinib treatment. Grade 3/4 neutropenia was observed in 18 patients (15%), and Grade 3/4 gastrointestinal disturbances were noted in 14 patients (12%) [12]. Table 3 provides a summary of the toxicity data.

**Table 3:** Toxicity

Toxicity	Number of Patients (%)
Grade 1/2 Neutropenia	35 (28.5%)
Grade 3/4 Neutropenia	18 (15%)
Grade 1/2 Gastrointestinal Disturbances	25 (20.3%)
Grade 3/4 Gastrointestinal Disturbances	14 (11.4%)
Hepatotoxicity	5 (4.1%)
Rash	10 (8.1%)

Grade 1/2 neutropenia: 35 patients (28.5%) experienced mild to moderate neutropenia. Grade 3/4 neutropenia: 18 patients (15%) experienced severe neutropenia. Grade 1/2 gastrointestinal disturbances: 25 patients (20.3%) had mild to moderate gastrointestinal disturbances. Grade 3/4 gastrointestinal disturbances: 14 patients (11.4%) had severe gastrointestinal disturbances. Hepatotoxicity: five patients (4.1%) experienced hepatotoxicity, which is a known side effect of imatinib. Rash: 10 patients (8.1%) developed a rash, a common side effect of TKIs.

## Discussion

This retrospective study provides valuable insights into the outcomes of imatinib dose escalation in CML patients treated in a resource-constrained setting. Our findings demonstrate that dose escalation to 600 mg or 800 mg of imatinib can achieve meaningful clinical responses in patients who show suboptimal response or progression on the standard 400 mg dose [13]. These results are particularly relevant for LMICs, where financial and logistical barriers often limit access to second-line TKIs, such as dasatinib and nilotinib.

The observed clinical responses to dose escalation are significant, especially considering the challenges faced in LMICs. In such settings, the high cost and limited availability of newer second-line TKIs pose substantial hurdles. Imatinib, being more accessible due to its longer presence in the market and availability in generic form, offers a practical alternative when dose escalation is considered. Our study shows that a substantial proportion of patients who do not achieve optimal responses at the standard dose can benefit from a higher dose, thus extending the utility of imatinib in these populations.

Imatinib has been the cornerstone of CML treatment since its introduction, dramatically improving survival rates and transforming CML from a fatal disease into a manageable chronic condition. However, the economic disparities between high-income countries (HICs) and LMICs create a gap in the availability of newer, more potent second-line therapies. While dasatinib and nilotinib have shown superior efficacy in clinical trials, their high costs and the need for close monitoring make them less feasible options in resource-limited settings. Therefore, imatinib dose escalation emerges as a critical strategy to optimize patient outcomes in these regions. This approach not only makes the best use of the resources available but also aligns with the need for cost-effective and sustainable cancer care in LMICs.

Our study provides real-world evidence that dose escalation can be an effective strategy for managing patients with suboptimal responses to standard-dose imatinib. This is particularly crucial for patients in LMICs, where access to second-line TKIs is often limited. The decision to escalate the dose of imatinib rather than switch to a second-line TKI is influenced by multiple factors, including the financial burden on patients and healthcare systems, the availability of drugs, and the infrastructure for monitoring treatment. The significant clinical responses observed in our cohort suggest that dose escalation can bridge the gap in treatment options and provide a viable pathway to disease control in resource-constrained environments.

However, it is important to contextualize these findings within the broader landscape of CML treatment. Previous studies have reported that switching to a second-line TKI can lead to better PFS outcomes compared to dose escalation of imatinib [14]. For example, Hochhaus et al. found that patients who switched to dasatinib or nilotinib after failing imatinib had a three-year PFS rate of 80% [15]. In contrast, our study observed a three-year PFS rate of 67% with dose escalation. While this suggests that second-line TKIs may offer superior outcomes, it is critical to recognize that in settings where these options are not readily available, dose escalation remains a viable and cost-effective strategy. The lower PFS rate in our cohort might reflect the inherent differences in patient demographics, treatment access, and monitoring capabilities between HICs and LMICs.

Moreover, the implications of dose escalation extend beyond immediate clinical outcomes. The long-term management of CML relies heavily on sustained disease control, with deep molecular responses being a key goal of therapy. While second-line TKIs have been associated with higher rates of deep molecular responses, our study highlights that dose escalation of imatinib can still achieve meaningful responses in a significant proportion of patients. This is particularly relevant in LMICs, where the ability to maintain long-term treatment without financial catastrophe is a critical consideration. The observed efficacy of dose escalation, coupled with the relatively favorable safety profile, supports its use as a practical alternative in contexts where second-line options are either unavailable or unaffordable.

The safety profile of high-dose imatinib observed in our study was consistent with previous reports, with hematologic and gastrointestinal toxicities being the most common adverse events [16]. Notably, the incidence of severe toxicities was relatively low in our cohort, which may be attributed to the cautious approach of gradual dose escalation and vigilant monitoring. This finding is encouraging as it supports the feasibility of using higher doses of imatinib even in settings with limited resources for managing severe toxicities. The ability to manage these toxicities with available resources is a testament to the adaptability of healthcare providers in LMICs, who often operate in environments with limited access to advanced supportive care.

Nevertheless, the study has certain limitations that must be acknowledged. The retrospective design inherently introduces the potential for selection bias, and the relatively small sample size limits the generalizability of our findings. Additionally, the absence of molecular response data restricts our ability to fully assess the long-term efficacy of dose escalation in achieving deep molecular responses, which are increasingly recognized as critical for sustained disease control. Furthermore, the lack of a direct comparison group within our study precludes definitive conclusions about the efficacy of dose escalation versus switching to second-line TKIs. While cross-trial comparisons suggest that second-line TKIs may offer better outcomes, our study highlights the importance of considering dose escalation in specific contexts, particularly in resource-constrained environments where switching therapies might not be an option.

Future research should focus on prospective studies that compare dose escalation with switching to second-line TKIs, particularly in LMIC settings. Such studies would provide more robust evidence on the efficacy and safety of these approaches, helping to inform treatment guidelines that are more attuned to the realities of healthcare in resource-limited environments. Moreover, the inclusion of molecular response data in future studies would be invaluable in assessing the long-term benefits of dose escalation and its role in achieving the ultimate goal of treatment-free remission.

In conclusion, while the landscape of CML treatment continues to evolve with the advent of newer TKIs, imatinib dose escalation remains a relevant and practical option in LMICs. Our study underscores the potential of dose escalation to provide meaningful clinical benefits in settings where access to second-line therapies is limited. This approach not only addresses the financial and logistical challenges faced by patients and healthcare systems in LMICs but also highlights the need for treatment strategies that are both effective and sustainable in diverse healthcare environments. As the global oncology community strives to ensure equitable access to cancer care, it is essential that we continue to explore and validate treatment approaches that are adaptable to the varied contexts in which patients live and receive care.

## Conclusions

In summary, while second-line TKIs may provide superior PFS outcomes, our findings underscore the potential of imatinib dose escalation as a practical and cost-effective approach in settings where alternative therapies are not feasible. Future studies, ideally prospective and including larger, more diverse patient populations, are needed to validate these findings and further explore the role of dose escalation in the management of CML in LMICs.
